# The Epidemiology of Hospital-Referred Head Injury in Ardabil City

**DOI:** 10.1155/2017/1439486

**Published:** 2017-02-01

**Authors:** Esmaeil Farzaneh, Ghasem Fattahzadeh-Ardalani, Vahid Abbasi, Fariba Kahnamouei-aghdam, Behnam Molaei, Elham Iziy, Habib Ojaghi

**Affiliations:** ^1^Faculty of Medicine, Ardabil University of Medical Sciences, Ardabil, Iran; ^2^Department of Biology, Faculty of Sciences, Islamic Azad University, Science and Researches Branch, Tehran, Iran; ^3^Traditional and Complementary Medicine Research Center, Sabzevar University of Medical Sciences, Sabzevar, Iran

## Abstract

*Background and Purpose*. Trauma is the leading cause of death for youth in developing countries. Given the prevalence of head trauma (HT) in society and its complication and burden, the epidemiologic study of head trauma is necessary and is the main aim of this study.* Materials and Methods*. This retrospective population-based survey describes the epidemiology of head injury in a defined population in Ardabil city. It includes all 204 patients with head injury referred to the University Hospital of Ardabil, Iran, during 2013-2014. Data were collected by a checklist and analyzed by statistical methods in SPSS.19. Significance level *p* < 0.05 was considered.* Results*. Of all registered cases, 146 (71.6%) were male and the rest of them were female. Most of HT patients lived in Ardabil city (60.8%). The mean age of patients was 22.6 ± 25.9 and most of victims were young. 24.5% of traumatic patients have injuries in severe to critical level (grade 3-4). The most cause of trauma was accidents (41.7%). Most of injuries occurred in night (55.9%) and in summer season (42.2%). Causes were traffic accident in 41.7%.* Conclusion*. Results showed that the leading cause of head trauma especially in the warm seasons is accidents and so, designing programs to reduce road accidents can dramatically reduce the rate of trauma in the future in Ardabil province.

## 1. Introduction

Today, trauma is the main cause of mortality, hospitalization, and disability in society in all age groups which leads to about 16000 deaths daily in society. Among all types of trauma, head injury is one of the most important causes of death in patients under 25 years and responsible for one-third of total deaths caused by trauma [1].

Each year, nearly 50000 people in United States die after a head trauma that most of them experience lifelong disability associated with brain injury [2].

In developed countries, the rate of deaths caused by head injury was about 21% in first month and this rate rises to 50% in developing countries [1].

In developing countries, trauma is the leading cause of death among teens and youths and also leading cause of disability and health related economic losses in most of such countries [3]. Trauma literally means the damage and injury, and head injury was defined as physical damage to the brain or skull caused by external forces [4, 5], and in terms of medical science, trauma referred to any blunt and penetrating injury or damage to body organs which occurred intentionally or unintentionally due to external factors including traffic trauma (accident), poisoning, falls, drowning, and other mechanisms [6, 7].

Many studies showed that brain damage is classified to primary and secondary head injury. The primary head injury is the primary damage that causes brain injury, such as bruising of the brain, crush, and rupture of blood vessels. Secondary damage within a few hours to several days after the primary injury is caused due to brain swelling and bleeding [8]. Unlike other parts of the body that dilated damage skin by causing swelling, damaged skull can never compensate for inflation components of the brain by causing expansion. So any bleeding or swelling inside the skull increased the volume of content in the nonreaction chamber and led to increased pressure within the brain and this pressure decreased blood flow, oxygen levels, and the accumulation of waste products in the brain [9].

Given the importance of head trauma and concussion consequences including loss of consciousness and death that follows [10], leading to serious decisions and health programs in the country and its contributes to trauma-associated morbidity and mortality and costs in the health care system, epidemiologic study of this common injury is necessary [5].

Head trauma is a rare etiology for intracranial aneurysms and estimated that only 1% of intracranial aneurysms are due to head trauma [11].

For doing full health programs and effort for reducing rate of risk to the head trauma and also need for having information and statistics about location of event and also lack of exact statistics about head trauma in Ardabil province, this study was conducted to investigate the epidemiology of head trauma in Ardabil province from April 2013 to September 2014.

## 2. Materials and Methods

### 2.1. Study Area

Ardebil is an ancient city in Iranian Azerbaijan. Ardabil is the center of Ardabil province. At the 2011 census, its population was 564,365, in 156,324 families, where the dominant majority is ethnic Iranian Azerbaijanis. Ardabil is located on the Baliqly Chay River, about 70 km (43 mi) from the Caspian Sea, and 210 km (130 mi) from the city of Tabriz. It has an average altitude of 1,263 meters (4,144 ft) and total area of 18011 km^2^ (6.954 sq mi). Neighboring on the Caspian Sea and the Republic of Azerbaijan, this city is and has been of great political and economic significance throughout history, especially within the Caucasus region. It is located on an open plain 1,500 meters (4,900 ft) above sea level, just east of Mount Sabalan (4,811 m), where cold spells occur until late spring. The University of Ardabil has four teaching hospitals and one trauma registry center. The trauma center has 218 hospital beds with area 22366 square meters. Ardabil city is the best place for agriculture and animal husbandry. The city is not an industrial city but have some little factories in around of city.

This retrospective population-based survey describes the epidemiology of head injury in a defined population in Ardabil city. It includes all 204 patients with head injury referred to the University Hospital of Ardabil, Iran, during 2013-2014. In addition, all head injured patients admitted to other departments of the hospital (due to accompanying injuries) and all patients referred to the emergency room but discharged after assessment were also included in the review.

For data collection we used a checklist including 5 parts as follows: designed demographic and identifying patients included clinical characteristics, age, sex, and vocational status, information about the trauma, and expense information. Information on trauma was including severity, etiology, trauma season, and outcome of trauma that is the most important part of this research which is based on who have died or have been discharged after a short time or have been hospitalized because of the severity of the damage. In this study, the patients were classified according to the HISS and trauma severity was measured by Glasgow Coma Scale (GCS). GCS in various grades are mild (GCS: 14-15), moderate (GCS: 9–13), severe (GCSS: 5–8), and critical (GCS: 3-4). Finally the collected data were analyzed by statistical methods in SPSS.16. The significance level was set at *p* < 0.05.

## 3. Results

The medical records of 204 patients (146 men, 58 women, age 1–89 years) referred to the hospital after head injury were reviewed. Of all registered trauma patients, 146 (71.6%) were male and the rest of them were female. Of all patients 56.4% were single and the mean age of patients was 22.6 ± 25.9 (range: 1–89). Most of victims are young, 60.8% from Ardabil city, and 19.1% self-employed. The most numbers of injuries according to mechanism were traffic accidents (41.7%) (Table 1).

114 (55.9%) of all head injuries occurred at the night. Most of cases were in summer season (42.2%) and 47.5% have severity of trauma in level 1. The most common cause of injury in all cases was accidents (41.7%).

After admission, 54.9% of patients were entered to study, 39.7% discharged after treatment in a short time, and 5.4% (11 people) have died. The most common cause of trauma related to public transit and road accidents that in the spring and summer reach to their peak rate. Most of head injuries occurred in summer (42.2%) (Table 2).

Both of men and women were faced with collapse after accident that this rate is much higher in men than women (Table 3).

Half of our cases (112 people) sustained severe head trauma that required hospital admission and extended care, some with poor outcome (Table 4).

## 4. Discussion

Head trauma as a leading cause of disability and death in young people is a major problem in society and yet it is preventable in the health systems [10]. The leading cause of deaths for more than 50 to 70% of cases is due to brain damage [11]. In 1998, about 8.5 million people around the world died due to trauma [12]. In America, every year millions of people die due to head trauma which can cause significant costs on the economy and that about 500 thousand of them die due to the trauma caused by vehicles [13]. The results also showed that the most common cause of head trauma is accidents and then falling. These results are consistent with the findings of Beigzadeh et al.'s study [3]. Among all kinds of trauma, head trauma is always dangerous to humans. Head trauma is damage to the head caused by a foreign object [14]. But in full expression and clearer stating the most important characteristic for a strong head kick or head trauma can be loss of consciousness longer than ten minutes, broken bones, and convulsions after hit [15]. The most dangerous emergency head trauma is blow to the head, skull, and brain [10]. The most common cause of death in accidents is also head [16]. As far as the highest cause of death in people less than 24 years is attributed to brain injuries which annually impose million dollars costs to the healthcare system [17].

In this study, similar to the results of Kashan, Urmia, Guilan, and Rasht, most of trauma patients are men [1, 2, 18, 19]. Trauma ratio of men to women was 2.5 to 1 in this study. In other studies in Iran, this ratio fluctuated between 2.5 and 5 [2, 20–22]. In a study in America men make up 54.6% of patients [23], and in another study men with head trauma were 2.6 times more than women [24]. Because there would be more participation of men in activities of daily living environment away from home and they used vehicles more which with head trauma causes of road accidents are well justified.

According to results, there is a significant relation between seasons and traffic accidents as the main cause of trauma, such that this rate was higher in summer that can be related to widespread use of motorbike in the hot season and increased traffic agriculture and rural areas. These results are consistent with the findings of Ebrahimipour et al.'s study in Mashhad and inconsistent with the results of Kasmaei et al.'s study that spring is the most traumatic season and falls with 62% are the most mechanism of trauma [1, 5, 25].

In this study, the average age of the patients was 26 years and it can be concluded that hit on head is more common in young people. This finding is consistent with findings of other studies in Iran and other countries [21, 26–29]. In this sense, it can be concluded that because young people make up the community activist group so they have incurred more accidents and of course have been hit on head; therefore it is necessary to promote the necessary training to youth culture.

## 5. Conclusion

Relying on the obtained information it can be stated that head trauma accounts for the huge annual costs mainly due to road traffic accidents and reaches its maximum rate in the summer and spring seasons and given that half of those surveyed in the most severely damaged and have been hospitalized for some time, it can be said that if statistical analysis could place useful information at the disposal of researchers this will be useful work, because all health programs and making cultures to reduce accidents and attacks have to rely on good information. Authorities in this field should apply all their efforts to implement ideas to raise awareness in the community on the use of safety devices and so on. It is hoped that, in the coming years with doing projects, we see a decrease in brain injuries and risks by providing a system to road-user safety.

## Figures and Tables

**Table 1:** Demographic and basic variables of the patients in study.

Variables	*N* (%)
Place of residence	
Ardabil	124 (60.8)
Other places	80 (39.2)
Marital status	
Single	89 (43.6)
Married	115 (56.4)
Sex	
Male	146 (71.6)
Female	58 (28.4)
Occupation	
Employee	13 (6.4)
Self-employee	39 (19.1)
Others	152 (74.5)
Time of event	
Night	90 (44.1)
Day	114 (55.9)
Reference season	
Spring	64 (31.4)
Summer	86 (42.2)
Fall	29 (14.2)
Winter	25 (12.3)
Mechanism of trauma	
Traffic accidents	85 (41.7)
Falls	56 (27.5)
Invasion	10 (4.9)
Other causes	53 (26)
Severity of trauma	
Mild (Grade 1)	97 (47.5)
Moderate (Grade 2)	57 (27.9)
Severe (Grade 3)	28 (13.7)
Critical (Grade 4)	22 (10.8)

**Table 2:** Distribution of trauma patients by season and mechanism of trauma.

Seasons	Cause of trauma				
	Traffic accidents *n* (%)	Falls *n* (%)	Invasion *n* (%)	Other causes *n* (%)	Total *n* (%)
Spring	29 (45.3)	18 (25.1)	1 (1.6)	16 (25)	64 (100)
Summer	31 (36)	27 (31.4)	6 (7)	22 (25.6)	86 (100)
Fall	12 (41.4)	7 (24.1)	1 (3.4)	9 (31)	29 (100)
Winter	13 (52)	4 (16)	2 (8)	6 (24)	25 (100)
Total	85 (41.7)	56 (27.5)	10 (4.9)	53 (26)	204 (100)

**Table 3:** The frequency of trauma patients by sex and cause of trauma.

Cause of trauma	Sex		
	Male *n* (%)	Female *n* (%)	Total *n* (%)
Traffic accidents	60 (41.1)	25 (43.1)	85 (41.7)
Falls	41 (28.1)	15 (25.9)	56 (27.5)
Invasion	7 (4.8)	3 (5.2)	10 (4.9)
Other causes	38 (26)	15 (25.9)	53 (26)
Total	146 (100)	58 (100)	204 (100)

**Table 4:** Frequency of trauma patients by jobs and result of trauma.

Job	Outcome			
	Death *n* (%)	Discharge *n* (%)	Hospitalization *n* (%)	Total *n* (%)
Employee	1 (7.7)	2 (15.4)	10 (76.9)	13 (100)
Self-employee	4 (10.3)	18 (46.2)	17 (43.6)	39 (100)
Other	6 (4)	61 (40)	85 (56)	152 (100)
Total	11 (5.4)	81 (39.7)	112 (54.9)	204 (100)
